# Mitochondrial Dysfunction and Alpha-Lipoic Acid: Beneficial or Harmful in Alzheimer's Disease?

**DOI:** 10.1155/2019/8409329

**Published:** 2019-11-30

**Authors:** Sávio Monteiro dos Santos, Camila Fernanda Rodrigues Romeiro, Caroline Azulay Rodrigues, Alícia Renata Lima Cerqueira, Marta Chagas Monteiro

**Affiliations:** ^1^Pharmaceutical Sciences Graduate Program, Institute of Health Sciences, Federal University of Pará, Belém, Pará, Brazil; ^2^Faculty of Pharmacy, Institute of Health Sciences, Federal University of Pará, Belém, Pará, Brazil; ^3^Neuroscience and Cell Biology Graduate Program, Institute of Biological Sciences, Federal University of Pará, Belém, Pará, Brazil

## Abstract

Alzheimer's disease (AD) is a neurodegenerative disorder characterised by impairments in the cognitive domains associated with orientation, recording, and memory. This pathology results from an abnormal deposition of the *β*-amyloid (A*β*) peptide and the intracellular accumulation of neurofibrillary tangles. Mitochondrial dysfunctions play an important role in the pathogenesis of AD, due to disturbances in the bioenergetic properties of cells. To date, the usual therapeutic drugs are limited because of the diversity of cellular routes in AD and the toxic potential of these agents. In this context, alpha-lipoic acid (*α*-LA) is a well-known fatty acid used as a supplement in several health conditions and diseases, such as periphery neuropathies and neurodegenerative disorders. It is produced in several cell types, eukaryotes, and prokaryotes, showing antioxidant and anti-inflammatory properties. *α*-LA acts as an enzymatic cofactor able to regulate metabolism, energy production, and mitochondrial biogenesis. In addition, the antioxidant capacity of *α*-LA is associated with two thiol groups that can be oxidised or reduced, prevent excess free radical formation, and act on improvement of mitochondrial performance. Moreover, *α*-LA has mechanisms of epigenetic regulation in genes related to the expression of various inflammatory mediators, such PGE2, COX-2, iNOS, TNF-*α*, IL-1*β*, and IL-6. Regarding the pharmacokinetic profile, *α*-LA has rapid uptake and low bioavailability and the metabolism is primarily hepatic. However, *α*-LA has low risk in prolonged use, although its therapeutic potential, interactions with other substances, and adverse reactions have not been well established in clinical trials with populations at higher risk for diseases of aging. Thus, this review aimed to describe the pharmacokinetic profile, bioavailability, therapeutic efficacy, safety, and effects of combined use with centrally acting drugs, as well as report in vitro and in vivo studies that demonstrate the mitochondrial mechanisms of *α*-LA involved in AD protection.

## 1. Introduction

Alzheimer's disease (AD) is a chronic and progressive neurodegenerative disorder, impairing brain functions such as memory, thinking, and personality [1, 2]. AD is characterised by several neuropathological changes, which include cerebral atrophy, intense synaptic loss, and neuronal death, in regions of the prefrontal cortex and hippocampus that are responsible for cognitive functions [3, 4]. The mechanism that explains the pathogenesis of AD has yet to be fully elucidated [5, 6], but several hypotheses have been explored to explain this origin—hyperphosphorylation of the tau protein and *β*-amyloid peptide deposits (A*β*) are among those accepted in the scientific milieu [6, 7]. The accumulation of A*β* activates cells of the immune system, such as astrocytes, activated microglia, and macrophages. In turn, the activated cells promote the loss of regulation of the inflammatory response, inflammation, and oxidative stress state, as well as increase the production of A*β*, creating a vicious cycle that continues during the life of the person with AD [8, 9].

Oxidative stress in AD can be validated by changes in the brain, blood cells, and biological fluids, associated with increased malondialdehyde (MDA), lipid hydroperoxides and isoprostanes, thiobarbituric acid reactive substances (TBARS), nitric oxide synthase, and reactive oxygen species (ROS) in AD patient samples, suggesting a systemic imbalance of redox status [10–12]. Oxidative damage to brain tissue can be explained by the fact that this tissue is more susceptible to oxidative stress due to high oxygen consumption and low regenerative capacity, high polyunsaturated fat content, and low antioxidant concentration [13, 14]. In this sense, several studies show that high levels of biomarkers of oxidative damage, such as MDA, 4-hydroxynonenal (HNE), and F2-isoprostanes detected not only in brain tissues but also in fluids and peripheral tissues, are associated with worse AD prognosis [13, 15, 16]. Then, the evaluation of these oxidative stress and tissue damage markers could serve as an indicator of progression and severity of this pathology [10, 17–19].

Biomarkers of oxidative stress and antioxidants could help in the prediction of clinical outcomes and possible benefits in therapy. However, available data do not show sufficient evidence for the use of biomarkers so far described as predictors of severity or clinical outcomes in AD [20]. On the other hand, the balance of antioxidant capacity reflects cognitive function [19], reaffirming the crucial role of antioxidant defence pathways against ROS-induced damage. The main source of ROS is mitochondria, and the dysfunction of this organelle in neurons may be one of the initiating processes of neurodegeneration [13]. Mitochondrial damage, such as mitochondrial disruption, can cause astrocytes to increase matrix production due to calcium or ROS release, consequently activating caspases and generating cell death [21]. Mitochondrial dysfunction may be key in the process of AD pathogenesis, as this organelle has a fundamental role in bioenergetic modulation in the cell. Conversely, current studies are still unable to confirm this hypothesis; knowing the mechanisms of AD may direct a treatment to delay or prevent this process.

Overall, recent reviews also report that other natural products, such as flavonoids, polyphenols, alkaloids, and glycosides, exhibit neuroprotective mechanisms, due to changes in the expression of transcription factors, in signalling pathways, as well as the activation of autophagy mechanisms, among others [17, 22–24]. In this context, alpha-lipoic acid (*α*-LA) is a naturally occurring molecule, with antioxidant and anti-inflammatory properties [25, 26], which plays several roles in the pathogenesis of neurodegenerative diseases, such as AD, and acts as a neuroprotective agent [27]. Alpha-lipoic acid has been largely used in some diseases that have oxidative stress as the main cause in pathological processes. The antioxidants properties of *α*-LA have shown benefits in peripheral neuropathies, as well as in nerve injuries and diabetic neuropathy [28, 29]. Moreover, *α*-LA increases the production of acetylcholine [30], inhibits the production of free radicals [31], and promotes the downregulation of inflammatory processes [32]. Studies have shown that patients with mild AD who were treated with *α*-LA showed a slower progression of cognitive impairment [27, 32, 33], though current drug therapy is only aimed at controlling the symptoms, modulating neurotransmitter levels, and is not effective in delaying the production of the disease [34]. Therefore, this review provides an overview of molecular mechanisms in mitochondrial function in the AD context, as well as pharmacokinetic and therapeutic safety parameters of *α*-LA.

## 2. Role of the Mitochondria on the Pathophysiology of AD

Studies have suggested for a while that mitochondria are involved in primary and secondary cascades of AD pathogenesis. In the primary cascade, changes in energy metabolism are believed to be the beginning for changes in the A*β* precursor protein (A*β*pp) homeostasis, as some classical studies and current reviews reported that bioenergetic disturbances (such as glucose deprivation) would shift the processing of A*β*pp to the amyloidogenic route [35]. Regarding the secondary cascade, there are hypotheses concerning the interference of A*β* in calcium homeostasis, because the protein reduces the cell's ability to lower cytoplasmic calcium levels, leading to synaptic mitochondrial impairment and a reduction in ATP production [36]. In addition, another well-elucidated pathway is related to the aggregation of the mitochondrial protein amyloid beta-binding alcohol dehydrogenase (ABAD) to A*β*. This complex is already understood as a potential pathogenic pathway, because in a pathophysiological context, this aggregation seems to have induced the production of ROS and led to neuronal death [37]. Thus, more recent studies have mentioned the inhibition of ABAD as a therapeutic target in AD to reduce the toxicity mediated by the ABAD-A*β* complex [38].

Mitochondrial damage in the neurons of individuals with AD has been known for 25 years [39], and a classic study proposed the mitochondrial cascade for the origin of AD, where a person's mitochondrial DNA (mtDNA) determines basal mitochondrial function and mitochondrial durability. This could be the cause of AD being more frequent in the elderly, an epigenetic mechanism with mutations in specific mtDNA fragments, due to aging [40]. A recent study attributed mitochondrial dysfunction and consequent accumulation of ROS to increased insulin resistance in neurons of the cortex and hippocampus, thus favouring the progression of oxidative lesions in DNA—namely, 8-oxoguanine (8-oxoG)—as an accumulation of this DNA lesion has been found in the brain of patients with AD [41]. The accumulation of 8-oxoG in mitochondrial DNA induces dysfunction and impairs neuritogenesis [42].

Post-mortem investigations of the brains of AD patients showed a reduced number of mitochondria, with a simultaneous increase of mtDNA and mitochondrial proteins in the cytosol [43]. These changes expressed in AD may be related to oxidative damage in mtDNA, a genetic material that is poorly protected by stabilizing proteins and is associated with mitochondrial dysfunction and aging. Accordingly, mitochondrial dysfunction caused by oxidative damage in mtDNA is associated with changes in the number of oxidative phosphorylation subunits and abnormalities in the fission and fusion processes of mitochondria, as well as damage in carrier proteins. These mechanisms are suggested as initiators in the early AD process [44]. The main mechanism of oxidative damage repair in DNA is defined as the base excision repair (BER) pathway, which has decreased activity in brain tissue, both in the genetic material of the nucleus and in mitochondria. These evidences, based on the analysis of cortex and cerebellum samples from individuals with AD, point to the possibility of a relationship between low BER activity and a higher level of neuronal death induced by A*β* toxicity and neurofibrillary plaques. However, due to conflicting results in the literature, it is not clear whether reduced BER activity leads to the accumulation of mutations in mtDNA [45].

Another altered mitochondrial pathway in AD is related to the sirt3 protein, a member of the sirtuin family of proteins responsible for epigenetic regulation, chromatin integrity, regulation of metabolism, and longevity, as well as playing a role in aging [17]. Sirt3, the subtype present in neuronal mitochondria and in several other cell types, is localised in the internal membrane and mitochondrial matrix and nucleus, participates in regulation of ROS production, and modulates the phosphorylation of CREB and fatty acid metabolism [46]. Modifications in electron transport chain dynamics, as well as increased ROS production and an unbalance in mitochondrial fusion and fission processes, cause mitochondrial damage; these mechanisms are cyclically propagated with high levels of ROS causing damage to proteins and DNA, increased lipid peroxidation, and consequent tissue damage. The production of ROS is one of the mechanisms that leads to the accumulation of A*β*, which is one of the characteristics of AD [47]. In the pathology of AD, aggregates of both A*β* and tau protein affect mitochondrial function and contribute to increased ROS production. In contrast, a recent study concluded that mitochondrial alterations are not dependent on high levels of A*β* and tau in the early stages of the disease, although they contribute significantly to neurodegeneration caused by mitochondrial dysfunction in more advanced stages of AD [48].

Several mechanisms, pathways, and processes in AD have yet to be elucidated, while much has been evidenced in relation to neuronal damage caused by mitochondrial dysfunction. Processes, such as mitophagy and biogenesis of mitochondria, are impaired due to mitochondrial dysfunction [43]. In addition, dysfunctional mitochondria regulate inflammatory responses through the activation of inflammasomes, a multicomplex protein that comprises nucleotide-binding domain activation, leucine-rich-containing family, pyrin domain-containing-3 (NLRP3) and sites where pro-IL-1*β* and pro-IL-18 processing take place, generating the activation of IL-1*β* and caspase-1, which is a crucial mechanism in the pathology of AD [49]. From this knowledge, specific therapeutic targets for the improvement of mitochondrial dynamics in central nervous system (CNS) cells are under investigation, aiming to use these new agents as effective treatments for AD and other neurodegenerative diseases [50]. In this sense, molecules with antioxidant, anti-inflammatory, and neuroprotective potential, such as alpha-lipoic acid, with action characteristics on mitochondrial machinery, are promising strategies in the treatment of AD.

## 3. Pharmacokinetics and Effects of *α*-LA in AD

Alpha-lipoic acid (*α*-LA) has been widely used in pharmaceuticals and nutraceuticals, precisely because it has antioxidant and anti-inflammatory properties [51, 52]. The chemical activity of *α*-LA is mainly due to the dithiolane ring, whose sulphur atoms confer a high electron density to *α*-LA. These effects, along with the hydrophilic and lipophilic characteristics of the molecule [53], as well as its status as the most efficient agent among all antioxidants [54], support studies with supplementation models for metabolic diseases and neurodegenerative diseases, such as AD. *α*-LA is classified as an ideal neuroprotective antioxidant because of its ability to cross the blood-brain barrier and its uniform uptake profile throughout the central and peripheral nervous systems [33, 55]. Synthesised in the mitochondria of animal and vegetable cells, as well as in microorganisms, *α*-LA can also be obtained from the diet through the consumption of dark green leafy vegetables and meats [56].

Biosynthesis of *α*-LA occurs in small amounts in the mitochondria from octanoic acid [52, 57], a natural cellular process during the metabolism of fatty acids. In contrast, the industrial production of *α*-LA results from chemical synthesis, and this process requires toxic catalysts in addition to sulphur atoms [58]. Therefore, *α*-LA can be found as a dietary supplement, a racemic mixture composed of its R-*α*-LA and S-*α*-LA isomers, whose main doses are 50, 100, 200, 300, or 600 mg per day. [59]. Other studies show beneficial effects of *α*-LA at doses of 1200 and 2400 mg in humans and interestingly no side effects [60]. In addition, the isolated R-*α*-LA isomer is used at doses of 200 and 300 mg [61].

The R-*α*-LA isomer is unstable at temperatures above 49°C, while the racemic mixture remains stable at temperatures between 60 and 62°C. Pharmacokinetic studies conducted in healthy subjects found that the R-*α*-LA isomer has a higher level of absorption, while the isomer S-*α*-LA assists in this percentage, preventing the polymerisation of the R-*α*-LA form [62]. In this sense, the use of supplements with the racemic mixture is more viable with respect to the R-*α*-LA isomer [63].

For oral supplementation of *α*-LA, the compound is rapidly absorbed and eliminated by the renal route. Because of its amphiphilic character, *α*-LA is widely distributed throughout all body compartments, including the CNS [52]. *α*-LA presented a mean time to reach the maximum plasma concentration (*t*_max_) of 15 minutes and a mean plasma half-life (*t*_1/2_) of 14 minutes [63]. These pharmacokinetic values differ among from other studies, where *t*_max_ was reported to occur within 30 minutes after oral administration [63] (10 minutes in rats) and *t*_1/2_ was approximately 30 minutes. The short half-life of *α*-LA is a result of extensive extraction and hepatic metabolism, which reduces the bioavailability of the ingested dose to 30%, on average [64]. Multiple factors influence the bioavailability of *α*-LA, including food intake, which interferes with the absorption of *α*-LA, either as the racemic mixture or the isolated isomers. Thus, *α*-LA consumption is recommended 30 minutes before or 2 hours after food intake [62]. The high variation in pharmacokinetic parameters of *α*-LA has been reported in several studies [51, 52, 61–64] but has not been fully explained. Changes in physiological conditions, such as gastric absorption and hepatic perfusion, or drug-drug interaction and drug delivery system mechanisms, are some justifications for the variation but have yet to be fully elucidated [46].

Absorption of at least 93% of the administered dose of *α*-LA, demonstrated in a rat study, occurred in the gastrointestinal tract, including the stomach [65]. In the gut, *α*-LA is internalised by the cells through receptors called the Na^+^/multivitamin transporter (SMVT). In humans, the present human Na^+^/multivitamin transporter (hSMVT) is responsible for the transport of biotin and pantothenic acid, iodine ions, and racemic *α*-LA. However, *in vitro* evidence suggests the existence of more than one cellular transport mechanism involving other fatty acid transporters, such as the monocarboxylic acid transporter (MCT) [57]. In fact, the *α*-LA intestinal absorption seems to be quite variable due to the existence of *α*-LA transport means still not completely explained. This complex system of absorption and distribution to the tissues suggests the existence of several factors involved, for example, substrate competition and transcriptional, translational, and posttranslational regulatory mechanisms [65].

After its internalisation into the cell, *α*-LA is catabolised through *β*-oxidation or enzymatic reduction to dihydrolipoic acid (DHLA), which together with *α*-LA forms two molecules with a high antioxidant capacity [56], generating a potential of reduction of -0.32 V [65]. The reduction of *α*-LA to DHLA occurs under the action of enzymes, such as mitochondrial dihydrolipoamide dehydrogenase, cytoplasmic or extracellular glutathione reductase, and cytoplasmic thioredoxin reductase [66]. *β*-Oxidation is the main metabolic pathway of *α*-LA, resulting from oxidation of the carbon side chain, generating several metabolites [67]. In addition, *α*-LA is alkylated by S-methyltransferases, and thus, only 0.2% of the administered dose is excreted unchanged in the urine [46]. In different species, 12 *α*-LA metabolites were identified [68], with at least five in the human species. 4,6-Bismethylthiohexanoic acid (BMHA) was identified as the major metabolite in urine samples from healthy volunteers after oral administration of *α*-LA, followed by lower concentrations of 6,8-bismethyl octanoic acid (BMOA) and 2,4-butyric acid (BMBA) [52]. To date, there are no studies demonstrating other routes of elimination of *α*-LA and its metabolites after supplementation.

*In vitro* studies, animal studies, and clinical trials have already demonstrated the pharmacokinetic and safety profiles of *α*-LA [61], interesting characteristics of the molecule, whether in its racemic form or the isomers. However, there are still few available data from studies in elderly individuals. Thus, this represents an inconvenient lack of accurate information in groups notably afflicted by diseases associated with aging, such as AD. Pharmacokinetic parameters of *α*-LA in individuals with AD are still scarce, considering the need for these types of studies related to the beneficial effects of *α*-LA in CNS pathologies. However, advances in the investigation of *α*-LA mechanisms linked to the processes associated with mitochondrial disorders and other cellular pathways associated with neurodegenerative disorders have been achieved in studies of all levels in recent years.

### 3.1. In Vitro Studies

Park and colleagues [69] reported the protective action of *α*-LA in glioma cells (C6) in a model of glutamate-induced cytotoxicity. In this work, cell cultures were incubated with *α*-LA—at a dose of 200 *μ*M—in a pretreatment scheme, one hour before the induction of glutamate cytotoxicity. As a result, the authors observed the suppression of apoptotic events, such as alteration of nuclear morphology and activation of caspase-3, and attenuation of stress markers in the endoplasmic reticulum. Glutamate-induced cytotoxicity is one of the possible causes of mitochondrial dysfunction, as it increases intracellular levels of calcium and thus activates several pathways, including apoptosis. Glutamate raises oxidative stress by increasing ROS production and glutathione (GSH) depletion *per se*, leading to cellular damage. In this sense, *α*-LA was effectively able to suppress cytotoxic effects in astroglial cells.

Dinicola and colleagues [53] performed a study on human neuroblastoma SK-N-BE cells treated with *α*-LA at a concentration of 0.5 mM for 24 hours. This work demonstrated the epigenetic regulatory activity of *α*-LA on the expression of the IL-1B and IL-6 genes responsible for the coding of interleukins IL-1*β* and IL-6, respectively. The authors investigated DNA methylation as a possible mechanism for the regulation that *α*-LA exerts on the gene regions. In this sense, they found lower mRNA levels of both genes in the *α*-LA group compared to the untreated group. The detection and quantification of IL-1*β* and IL-6, performed by an Enzyme-Linked Immunosorbent Assay (ELISA), also revealed lower levels in the culture supernatant of *α*-LA-treated cells compared to the supernatant untreated cells. Additionally, in treated cells, DNA methylation levels were inversely correlated to the levels of mRNA encoded for IL-1*β* and IL-6. This same group conducted another study on the treatment of ovarian cells with *α*-LA and obtained similar results on the downregulation of IL-1*β* and IL-6. The data obtained in this study suggest that *α*-LA modulates these proinflammatory cytokines (directly or indirectly) related to several pathological processes, including neurodegeneration [70].

Baeeri et al. [71] used rat embryonic fibroblast cells to evaluate the antioxidant effects of *α*-LA on senescence and cell cycle, oxidative stress, and inflammatory stimuli. These cells were submitted to *α*-LA concentrations of 1 to 1000 *μ*M for 24 hours, and subsequently, oxidative stress, cell viability, cellular senescence biomarkers, and inflammatory markers were evaluated. The authors described the EC50 of *α*-LA, defined in this study at 947 *μ*M, even though the highest dose evaluated (1000 *μ*M) did not cause a toxic effect. *α*-LA reduced the levels of oxidative stress parameters, such as lipid peroxidation, ROS, ferric reducing antioxidant power (FRAP), and total thiol molecules (TTS). Senescence markers were also reduced with *α*-LA treatment, with lower levels of *β*-galactosidase, as well as reduced levels of apoptosis and necrosis, which presented values of 36% and 15.7%, respectively. Additionally, *α*-LA had an effect on caspases-3 and -9, reducing the activity of these apoptosis-promoting molecules to basal levels. The inflammatory parameters TNF-*α*, IL-1*β*, IL-6, and NF-*κ*B showed reduced levels in cells treated with *α*-LA, maintaining levels similar to those of control cells. These results point out the benefits of *α*-LA on cellular senescence, in addition to oxidative stress and inflammatory processes. Thus, *in vivo* studies are required to verify the interaction of *α*-LA with endogenous enzymes and biochemical pathways of cellular senescence.

### 3.2. Animal Studies

Mahboob et al. [72] demonstrated *α*-LA activity on the cholinergic system in an animal model of aluminium-induced neurotoxicity, associating memory, and learning effects dependent on the hippocampus and the amygdala. The BALB/c mice from this study were divided into control, aluminium, and aluminium+*α*-LA groups. An *α*-LA dose of 25 mg/kg/day was administered intraperitoneally for 12 days. After this period, the authors verified the expression of muscarinic (M1–M5) and choline acetyltransferase (ChaT) receptors in these regions of the brain using RT-PCR, PCR, and tissue histopathology and complemented their investigation with an *in silico* docking model of *α*-LA in two types of these receptors (M1 and M2). The results of this study showed positive effects of *α*-LA on applied behavioural tests, as well as the reversal of neurodegeneration evidenced by histopathology. *α*-LA increased the expression of M2 muscarinic receptors in the hippocampus and M1 and M2 in the amygdala, in addition to ChaT expression in both regions. In the *in silico* assays, *α*-LA showed a high affinity for M1 and M2 receptors compared to acetylcholine, a muscarinic receptor agonist. In this evaluation, the R-*α*-LA and S-*α*-LA isomers were coupled to the M1 and M2 receptors, where the R-*α*-LA isomer showed a higher mean binding affinity for both receptors. Together, these results showed the effects of *α*-LA on memory and learning, improving these conditions in a neurodegeneration model. However, these data should be carefully analysed, since one of the characteristics of *α*-LA involves chelating metals. Therefore, such effects could be the result of a direct *α*-LA mechanism on the agent used to simulate the disease model, which does not represent a natural physiological mechanism or part of the pathological process evaluated in a real situation.

An animal study conducted by Zhang et al. [73] aimed to verify whether *α*-LA could improve the state of tauopathy by investigating if the inhibition of ferroptosis, a mechanism associated with pathology, could reverse cognitive impairment in the AD model. P301S transgenic mice received *α*-LA treatment at doses of 3 and 10 mg/kg/day intraperitoneally for 10 weeks. In this work, behavioural parameters, immunohistochemistry, fluorescence, and expression of factors essential for the metabolism of iron and cell pathways involved in tau protein dysfunction, as well as synaptic loss and apoptosis, inflammation, and oxidative stress, were evaluated. The authors reported positive effects of *α*-LA on several key points in the pathophysiology of tauopathy, one of the disorders associated with AD. In this sense, *α*-LA could protect neurons from toxicity caused by hyperphosphorylation of tau, acting through mechanisms such as inhibition of ferroptosis. In addition, *α*-LA decreased the high expression of calpain 1, cleaved caspase-3, and increased levels of neuronal nuclear protein (NeuN) and synaptophysin (SYP), inhibiting neuronal loss and synaptic dysfunction. These results suggest that *α*-LA acts on these apoptotic signalling pathways, leading to improved cognitive function and attenuation of neurodegeneration.

Tzvetanova and colleagues [74] tested the neuroprotective potential of *α*-LA in an animal model of scopolamine-induced dementia. Male Wistar rats were treated with an intraperitoneal 30 mg/kg/day dose of *α*-LA, intraperitoneally, for 11 days. After the end of the treatment, the authors evaluated the memory and learning of the animals, as well as parameters of oxidative stress in the collected brain tissue. The experimental model with scopolamine followed dementia-like results similar to AD in the groups treated with this drug, evidenced in the behavioural tests, with impairment in memory and learning. Thus, increased oxidative stress was observed throughout the brain tissue, including in areas responsible for memory and learning, such as the cortex, hippocampus, and striatum. In these tissues, lipid peroxidation levels increased, as did GSH, catalase (CAT), and superoxide dismutase (SOD) levels. Conversely, supplementation with *α*-LA reversed this oxidative profile, with GSH valuesin the cortex close to the values of the control group and reduced GPx in all tissues analysed. In the *α*-LA group, the levels of catalase were kept close to the values of the control group, different from the scopolamine group. In addition, the authors observed a slight reduction in SOD values in the animals treated with *α*-LA, compared to the scopolamine group. These findings reaffirm the *α*-LA profile on oxidative stress in a dementia model, suggesting benefits in this aspect for other diseases associated with neurodegenerative processes.

### 3.3. Human Studies

Spain and colleagues [75] showed the benefits of *α*-LA supplementation as a racemic mixture in multiple sclerosis in people aged 40 to 70 years. In this double-blind, randomised, placebo-controlled study, *α*-LA was used by 27 patients at a dose of 1200 mg/day given orally for two years. The comparison between the *α*-LA and placebo groups showed differences in the percentage change brain volume (PCBV) where 68% less cerebral volume loss was observed in *α*-LA patients. Despite the PCBV benefits, some patients had altered biochemical parameters (increased alkaline phosphatase), renal failure, and glomerulonephritis. These findings, although not clearly attributed to *α*-LA, suggest caution with this dose in future investigations.

Fiedler and colleagues [76] carried out a study comparing the pharmacokinetic parameters in a group of healthy individuals and a group of individuals with multiple sclerosis. A single 1200 mg dose of *α*-LA was administered to the subjects, and blood samples from individuals were collected at 1, 2, 3, 4, 24, and 48 hours after *α*-LA administration. In this study, the age of the subjects ranged from 50 to 58 years and the mean *α*-LA plasma concentrations (*C*_max_) in the healthy and multiple sclerosis groups were 1985.74 and 1894.52 ng/mL, respectively. These authors confirmed that *α*-LA metabolism does not change, and they did not observe differences between the groups, indicating that the bioavailability of *α*-LA was maintained even in individuals affected by multiple sclerosis compared to healthy subjects.

Shinto et al. [77] conducted a randomised placebo-controlled clinical study in patients diagnosed with AD, where they assessed the benefits of *α*-LA combined with omega-3 fatty acids. In this evaluation, the authors reported a delay in the progression of cognitive and functional decline in patients who received *α*-LA and omega-3 fatty acids. Thirty-four patients received 600 mg/day of *α*-LA, in addition to omega-3 fatty acids, for one year. Unfortunately, the experimental design of this study did not evaluate an *α*-LA-only group, as little was illuminated about the additive or isolated effects of the combined treatment. Therefore, the exclusive evaluation of *α*-LA supplementation in patients with AD, which could provide pharmacokinetic data or molecular mechanisms of the drug in relation to the physiology of individuals with neurodegenerative processes, has yet to be described in clinical trials.

In contrast, a 12-month open label study with nine individuals (eight men and one woman) diagnosed with AD, who received standard treatment with cholinesterase inhibitors, was performed in Germany by Hager et al. [59] Based on their results, the authors suggest that treatment with *α*-LA would be a successful neuroprotective option in AD, at least as an adjuvant to standard treatment with acetylcholinesterase inhibitors. This work was the first to highlight the benefits of *α*-LA in the treatment of AD, despite a small number of patients and a nonrandomised design. However, there are no data on pharmacokinetic parameters described in this study, demonstrating only positive *α*-LA results in neuropsychological parameters, such as mini-mental state examination (MMSE) and an AD assessment scale, cognitive subscale (ADAS-cog).

A large number of *in vitro* and *in vivo* studies, as well as some clinical studies, have been carried out in recent years with the aim of evaluating the effects of *α*-LA on neurodegenerative processes. Several cellular pathways have been mentioned and even tested for the involvement of antioxidants, such as *α*-LA. However, most of these studies are limited to the use of combinations of two or more antioxidants, such as the study by Shinto et al. [77], which evaluated the effects of two antioxidants (*α*-LA omega-3) on individuals with AD. Sharman et al. [78] evaluated the effects of curcumin associated with several other antioxidants, among them, *α*-LA, in an AD animal model. These studies contribute to the limited knowledge on the action of *α*-LA as an antioxidant and an anti-inflammatory agent, but they do not elucidate the mechanisms of action involved in these processes, since they do not verify isolated *α*-LA activity in these disease models. The actions of *α*-LA seem to go deeper than scavenging ROS, reestablishing the reduced form of other antioxidants, and chelating metals. Thus, studies are needed to apply *α*-LA in specific models of AD aimed at elucidating molecular mechanisms related to treatment and reversal of damage in neurodegenerative disorders.

## 4. Molecular Mechanisms of *α*-LA in Neurodegeneration

In recent years, the effects of *α*-LA on the pathogenesis and/or progression of neurodegenerative diseases, such as Parkinson's disease, multiple sclerosis, and AD, have been reported [51, 57]. In AD, the neurodegeneration process seems to start from mitochondrial dysfunction and consequent energy deficiency in neurons, in the early stages of the disease [79]. Additionally, *α*-LA reduces the migration of T lymphocytes and monocytes into the central nervous system [76], a mechanism that contributes to a microenvironment with fewer inflammatory factors. Thus, the effects of *α*-LA are described in several recently published studies, which present it as a molecule associated with the genomic regulation of antioxidant and anti-inflammatory pathways [62]. The mechanisms of *α*-LA on the cellular machinery include several activities attributed to its oxidised and reduced forms (dihydrolipoic acid (DHLA)), both being active as antioxidants and anti-inflammatory agents [57, 62]. In the reduced form, DHLA exerts its antioxidant effect by donating electrons to prooxidants or oxidised molecules [54]. In fact, *α*-LA's main antioxidant defence activity is its ability to reestablish the reduced forms of other endogenous antioxidants, such as vitamins C and E, in addition to elevating the intracellular levels of glutathione and ubiquinone [56, 62]. However, more and more *α*-LA studies show the complexity of the mechanisms of action attributed to this molecule, in addition to mechanisms already extensively described in the literature. Current findings on *α*-LA mechanisms show that it acts indirectly on the activation of signal transduction pathways [80] through interactions with second messengers, such as cyclic adenosine monophosphate (cAMP). The increase of cAMP caused by *α*-LA inhibits the release of proinflammatory cytokines, such as IL-2, IFN-*γ*, and TNF-*α*. Moreover, cAMP induces IL-10 production [76], an anti-inflammatory cytokine responsible for inhibiting the production of various inflammatory mediators induced by lipopolysaccharide (LPS). In addition, IL-10 is responsible for inhibiting the expression of cytokine receptors and histocompatibility complex II (MHC-II) receptors, chemokines, and microglial adhesion molecules [81].

The ability of *α*-LA to regulate certain genes, such as genes that encode nuclear factors (nuclear factor erythroid 2-related factor-2 (Nrf2) and NF-*κ*B) [53], has been reported previously; thus, *α*-LA is considered a pleiotropic molecule. The action of *α*-LA on NF-*κ*B affected the expression of several inflammatory mediators, such as PGE2, COX-2, iNOS, TNF-*α*, IL-1*β*, and IL-6 [82]. NF-*κ*B activation involves phosphorylation and ubiquitination under conditions of stress and inflammatory response. One of the mechanisms of regulating NF-*κ*B involves its repression, which is carried out by SIRT1 via deacetylation. In this sense, the action of *α*-LA on SIRT1 may be one of the means of regulation in posttransduction control pathways [71]. Alternatively, the molecular mechanisms of gene regulation appear to result from hypermethylation of DNA in the region of gene promoters, as in the case of the 5′ flanking regions of IL-1*β* and IL-6. Thus, mRNA is downregulated, and consequently, synthesised protein levels decrease [70]. However, detailed *α*-LA mechanisms on direct or indirect pathways of epigenetic regulation have not been elucidated yet. Thus, a better understanding this action of *α*-LA could leverage research for more effective therapies in AD. Epigenetic mechanisms, such as DNA methylation dysfunction, histone acetylation, and regulation of miRNAs, play a crucial role in the pathology of AD [83]. In this sense, epigenetic interventions could also be useful in preventing the disease, because in this case, factors in the microenvironment of the brain would be corrected through epigenomic modification [84].

The effects of *α*-LA on mitochondrial performance have already been described in previous studies to occur through several protective mechanisms. *α*-LA can act directly to prevent ROS production using the thiol group for redox regulation [85] and to stimulate enzymatic synthesis, such as frataxin which plays a role in the biosynthesis and assembly of the haem group in mitochondrial matrix proteins [86]. Indirectly, *α*-LA acts by improving the antioxidant system and stimulating mitochondrial biogenesis. Moreover, R-*α*-lipoic acid acts as a cofactor of mitochondrial enzymatic complexes and is therefore essential for energy production and metabolic regulation [87]. These protective routes make *α*-LA a substance classified in studies as a “mitochondrial nutraceutical.”

Among the pathophysiological processes of AD, some mechanisms of *α*-LA action on tau protein hyperphosphorylation and amyloidogenesis inhibition are known and described in the current literature [73, 88]. Treatment with *α*-LA promotes the control of kinases such as CDK5, GSK3*β*, and MAPK that, together with changes in iron metabolism, lead to protein hyperphosphorylation and other effects such as free radical increase and macromolecule oxidation [73, 89]. In P301S transgenic mice with alteration to tauopathy, the *α*-LA reduced tau protein levels by modulating activity of calpain 1 and possibly other kinases. Furthermore, in this study, inhibition of neuronal loss and ferroptosis attributed to *α*-LA action was also observed. These data show possible pathways of beneficial action of *α*-LA in AD, such as improvements in iron loading, lipid peroxidation, and inflammation in the CNS [73]. In inhibiting amyloid tangles, *α*-LA demonstrated direct activity on A*β* protein aggregates, destabilizing as interactions between lipoyl groups and through covalent connections with A*β* lysine residues. The action of *α*-LA is associated with the reduction of A*β* deposits and the destabilizing aggregate preforming of this protein [88].

An animal model of tauopathy provided evidence that mitochondrial function is affected in this condition of tau protein dysregulation and consequent formation of fibrillar aggregates in the CNS. This state influences ATP production in mitochondria, causing imbalance of energy metabolism and ROS formation, thus making the neuron more susceptible to increased oxidative stress. [90]. In this sense, *α*-LA could act directly on the restoration of mitochondrial function, promoting increased synthesis of mitochondrial enzymes such as frataxin [86] or as an enzymatic cofactor, restoring mitochondrial energy metabolism [87]. Furthermore, just as tau may influence mitochondrial function, oxidative stress caused by mitochondrial dysfunction may influence abnormal tau protein function. This mechanism seems to be deeply associated with the pathogenesis of AD [90]. In addition to the effects on tauopathies, *α*-LA in its reduced form (DHLA) showed significant protection against neurotoxicity to amyloid *β*-peptide and iron and hydrogen peroxide in a study of neuronal cell culture [91]. This evidence points to the benefits of *α*-LA over these mechanisms in the pathogenesis of AD, acting as a potential therapeutic agent in multiple cell pathways.

### 4.1. In Vitro Studies

**Figure 1:**
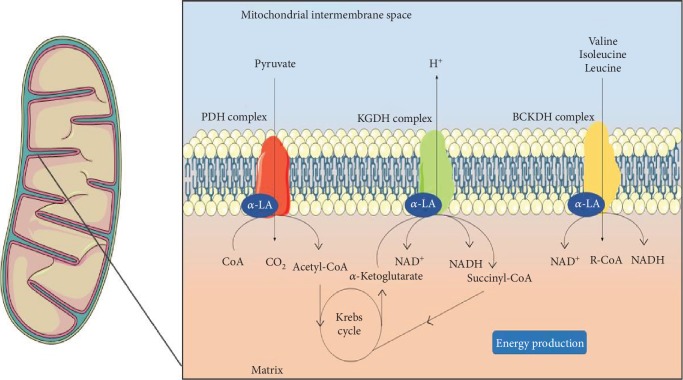
Physiologically, *α*-LA acts as a cofactor for enzymatic complexes during the oxidative phosphorylation process, such as pyruvate dehydrogenase (PDH), where *α*-LA residues are found in the dihydrofolipoamide acetyltransferase (E2) chain. Similarly activities occur with *α*-ketoglutarate dehydrogenase (KGDH) and branched chain *α*-ketoglutarate dehydrogenase (BCKDH) mitochondrial complexes. Thus, *α*-LA plays an important role in normal metabolic performance. This figure used elements from Servier Medical Art (https://www.servier.com).

An important *in vitro* study evaluated the effects of the association between coenzyme Q-10 and *α*-LA on human lymphocytes. The parameters analysed were DNA damage using the comet assay, mitochondrial membrane potential using flow cytometric readings of a cyanine dye capable of penetrating the mitochondria, and an intracellular ROS assay using flow cytometric analysis of intracellular DCFH-DA oxidation. The fluorescence reading indicated that the mitochondrial membrane potential was enhanced by the decreased ratio between the green monomers of JC-1 and red fluorescence of the J-aggregates at baseline. Regarding ROS analysis, the combination of coenzyme Q-10+*α*-LA significantly enhanced the oxidant-mediated increase in intracellular ROS, contrarily suggesting a prooxidant activity of these supplements [92]. *α*-LA also plays an important role in the metabolic performance of mitochondrial activity, as *α*-LA acts as a cofactor of pyruvate dehydrogenase (PDH), *α*-ketoglutarate dehydrogenase (KGDH), and branched chain *α*-ketoglutarate dehydrogenase (BCKDH) mitochondrial complexes. In addition, *α*-LA is reduced to dihydrolipoic acid by PDH and KGDH. These direct activities have already been reviewed from previous *in vitro* studies, as can be seen in Figure 1 [87]. In addition to the direct actions, the *α*-LA has presented in several studies the potential to improve the activity and recovery in mitochondrial dysfunction through indirect mechanisms, such as modulation of signalling and transcription. A study evaluated the expression of NAD-dependent protein deacetylase sirtuin-1 (SIRT1), NAD-dependent protein deacetylase sirtuin-3 (SIRT3), and coactivator of the peroxisome proliferator-activated receptor 1*α* (PGC-1*α*) in liver and bovine muscle cultures treated with resveratrol and *α*-LA. Cell cultures were treated for 60 minutes with 40 or 80 *μ*M of resveratrol and 30, 100, 300, or 1000 *μ*M of *α*-LA. In one group, a basal medium was used to mimic restricted nutritional conditions, and glucagon and epinephrine were used in the others to mimic the glycogenic state. Real-time PCR was performed to quantify the expression of mRNA for sirtuins and PGC-1*α*. *α*-LA, at the doses used, did not affect the expression of sirt1, whereas Forkhead box protein O3 (FoxO3) and isocitrate dehydrogenase mitochondrial (IDH2) in bovine liver cells were downregulated. The effects of *α*-LA on the expression of NAD-dependent protein deacetylase sirtuin-3 (SIRT3), Forkhead box protein O1 (FOXO1), 5′-AMP-activated protein kinase catalytic subunit alpha-1 (PRKAA1), glucose-6-phosphatase catalytic subunit (G6PC), and PGC-1*α* genes were dose-dependent. On the other hand, *α*-LA showed a positive effect on the expression of SIRT1 and SIRT3 in bovine muscle cells. Protein expression encoded by SIRT1 showed higher levels after *α*-LA treatment, especially in liver cells. The results allowed for the conclusion that the conditions induced in both cell types and the treatment with resveratrol and *α*-LA led to the positive expression of genes involved in the antioxidant response [93].

### 4.2. Animal Studies

**Figure 2:**
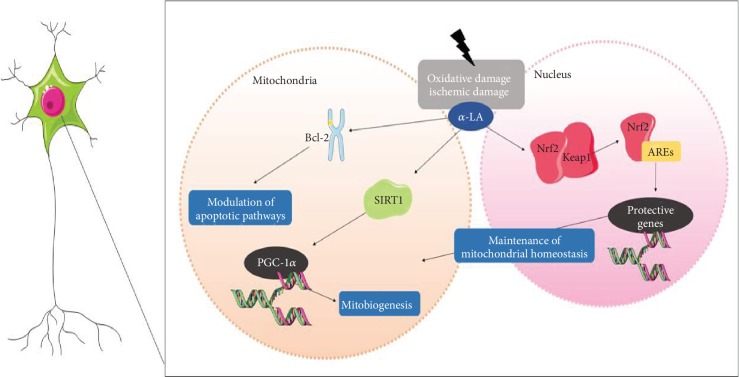
Cells treated with *α*-LA when subjected to oxidative or ischemic damage present higher expression of Blc-2 genes, modulating apoptotic pathways; sirt1 which induces peroxisome proliferator-activated receptor 1*α* (PGC-1*α*) and, therefore, mitochondrial biogenesis in the nucleus, positively regulates the nuclear factor erythroid 2-related factor-2 (Nrf2) pathway, improving the Nrf2 complex and antioxidant response elements (ARE), inducing the expression of genes that participate in mitochondrial homeostasis. This figure used elements from Servier Medical Art (https://www.servier.com).

The expression of SIRT1 was investigated in a study in rats that evaluated the neuroprotective potential of *α*-LA in a focal ischemia model. The groups were divided into sham-operated, permanent mean cerebral artery occlusion (pMCAO), and the group treated with *α*-LA (50 mg/kg) intraperitoneally 30 minutes before surgery. The parameters evaluated were neurological deficits from a blinded examiner's test to analyse the impairment of motor motion injury, infarct volume, cerebral oedema, immunohistochemistry of cells labelled for SIRT1 and PGC-1*α* over five regions of the induced injury, western blot, and qPCR to analyse the mRNA levels of SIRT1 and PGC-1*α* at 24 h after pMCAO. In the results of neurological deficit assessment, after 24 hours of pMCAO, a significantly reduced score was observed in the group treated with *α*-LA. In addition, the water content measured to assess cerebral oedema was reduced in this group, compared with the pMCAO group. Infarct volume was also lower in the *α*-LA group. Immunohistochemistry indicated a significant increase in the expression of SIRT1 and PGC-1*α* on the ischemic cortex, which corroborated with the western blot results. These data demonstrate the ability of *α*-LA to attenuate the lesions induced by cerebral ischemia [33]. It has been known that overexpression of SIRT1 activates the transcriptional activity of PGC-1*α* that also activates several transcriptional factors and increases mitochondrial activity [1]. A most recent study also investigated the neuroprotective effects of *α*-LA on an ischemic model and assessed the role of mitochondria via activation of Nrf2. The rats were randomly divided into a sham group, control group (MCAO+saline), *α*-LA-20 mg/kg MCAO group, and *α*-LA-40 mg/kg+MCAO group. Initially, parameters of infarct behaviour and volume were evaluated. Again, it was observed that *α*-LA reduces cerebral oedema and improves the neurological outcome in the MCAO model. In addition, antioxidant enzymes (SOD and GSH-Px) and malondialdehyde (MDA) were analysed by ELISA after 24 h of MCAO, which showed that the enzymatic activities were recovered and MDA was reduced in the *α*-LA-treated groups in a dose-dependent manner. These results signified the ability of *α*-LA to reverse the suppression of ischemia-induced antioxidant activity. Nuclear translocation of Nrf2 was assessed by immunofluorescence in primary cortical neurons. The cells were pretreated with the control and *α*-LA (at concentrations of 1 *μ*M, 10 *μ*M, or 100 *μ*M), and after 1 h, they were subjected to oxygen glucose deprivation (OGD) for 24 h. The results indicated that *α*-LA was able to modulate the activation and nuclear position of Nrf2 after the damage caused by MCAO. The ratio of nucleus/cytoplasmic Nrf2 was higher in the *α*-LA group 40 mg/kg, indicating that the activation of this factor also occurred in a dose-dependent manner [94]. The role of Nrf2 in mitochondrial function has been described in the context of neurodegeneration due to its role on the pathogenic processes in diseases [95]. NrF2 acts on redox homeostasis due to the regulation of target genes involved in the expression of antioxidant enzymes and on the expression of mitochondrial repair factors [96]. A recent study reported the activation of Nrf2 and subsequent binding to antioxidant response elements (ARE), in addition to decreased oxidative stress, *β*-amyloid (A*β*), and improved cognitive function in a mouse model of AD [97]. Therefore, this study suggests possible ways that *α*-LA could be inducing the overexpression of Nrf2 and promoting improved mitochondrial function in neurons. Mitochondria are also involved in the apoptosis process, especially in the context of neuronal damage. In this perspective, a study evaluated the influence of *α*-LA on the expression of the Bcl-2 apoptosis regulator (Bcl-2) in hippocampal subregions of rats submitted to inorganic arsenic. The animals were divided into three groups: the control group, the group that only received NaAsO_2_ (1.5 and 2.0 mg/kg bw), and the group that received NaAsO_2_+*α*-LA (1.5 mg and 70 mg/kg bw) and NaAsO_2_+*α*-LA (2.0 mg and 70 mg/kg bw). The TUNEL assay was performed to detect DNA fragmentation in cells; free floating immunohistochemistry, where apoptotic proteins were labelled, was used for cryocut coronal sections of the hippocampus; and western blot was used to determine the levels of apoptotic proteins in fresh hippocampal tissue. The results showed that the nuclei of the pyramidal and granular cells in the CA1 and CA2 regions (cornu ammonis), which received *α*-LA concomitantly, presented lower DNA fragmentation, compared to the control. Immunohistochemistry evidenced an intense expression of Bcl-2 in the hippocampus region of animals cotreated with *α*-LA and inorganic arsenic, similar to quantitative western blot results that enhanced the *α*-LA-inducing role in Bcl-2 expression, which was not dose-dependent [98]. Similarly, a most recent study investigated the protective role of *α*-LA against chlorpyrifos-induced toxicity, which rescued cells by modulating apoptotic pathways [99]. Another study investigated the protection of *α*-LA, through mitochondrial pathways, against cadmium-induced toxicity [100]. These studies conducted their investigation in the liver and kidney, respectively, thus lacking current mitochondrial investigations in neuronal rescue after toxicity. These processes are summarised in Figure 2.

## 5. Effects of *α*-LA When Combined with Central-Acting Agents

The effects of the association of *α*-LA with other substances acting on the CNS require a more in-depth description of the mechanisms observed in vitro, animal models, and human studies for a safe relation to be established, thus establishing the real clinical relevance of these findings.

### 5.1. Animal Studies

There is evidence that *α*-LA contributes to several treatments conducted with central-acting agents and in peripheral nerve cells. A study in mice evaluated the combination of *α*-LA and clozapine in the reversal of ketamine-induced symptoms similar to schizophrenia. Symptoms were induced for seven days, and from the eighth day, the ketamine group and the control group received *α*-LA (100 mg/kg), clozapine 2.5 or 5 mg/kg, or *α*-LA+clozapine 2.5 mg or 5 mg. The parameters evaluated regarding the symptoms were prepulse inhibition (PPI) of the startle locomotor activity, social preference, and Y-maze. In addition, oxidative stress parameters were evaluated in the prefrontal cortex (PFC), hippocampus, and striatum. In the PFC, brain-derived neurotrophic factor (BDNF) was determined. The results showed that the concentration of *α*-LA+clozapine 2.5 mg was able to revert the behavioural and oxidative stress parameters without causing harm. However, the concentration of *α*-LA+clozapine 5 mg induced motor impairment in the animals. Both concentrations significantly reversed the BDNF deficiency promoted by ketamine. Another relevant result was the reduction of ketamine-induced hyperlocomotion that was promoted by the association of *α*-LA and clozapine [101]. In another animal model study, *α*-LA was combined with chlorpromazine to observe its effects on schizophrenia induced by ketamine. Rats were treated for 10 days with saline, *α*-LA 100 mg/kg or chlorpromazine (1 mg/kg, 5 mg/kg), or a combination of *α*-LA+chlorpromazine after ketamine treatment. The behavioural and oxidative stress parameters evaluated were the same as those of the study by Vasconcelos et al. [101]. The authors observed that the combination of *α*-LA+chlorpromazine elevated the locomotor activity of the animals to the levels of the control, possibly by reducing the inhibitory effect of ketamine on N-methyl-D-aspartate (NMDA) cortical receptors [102]. The improvement in the oxidative profile was described by an earlier study in humans that evaluated the effects of *α*-LA on the antioxidant defence of patients on drug therapy for schizophrenia. However, these drugs used by participants were not mentioned by the authors, and specific interactions between *α*-LA and these antipsychotic medications have not been described [103]. Desvenlafaxine is a serotonin-norepinephrine reuptake inhibitor (SNRI) used for the treatment of depression [104]. In a study conducted in a neuroendocrine model of depression in animals, female rats were treated with corticosterone, corticosterone+desvenlafaxine (10 or 20 mg/kg), *α*-LA (100 or 200 mg/kg), *α*-LA (100 mg/kg)+desvenlafaxine *α*-LA (200 mg/kg), desvenlafaxine (100 mg/kg), or *α*-LA (200 mg/kg)+desvenlafaxine (20 mg/kg). The cognitive evaluation was performed using the tail suspension test (TST), social interaction (SIT), new object recognition (NOR), and Y-maze. In addition, the activity of the enzyme acetylcholinesterase (AChE) was measured in the prefrontal cortex, hippocampus, and striatum. The results demonstrated that the associated schemes were able to reverse short-term and long-term memory deficits and control the increase of AChE in PFC, HC, and ST, effectively with the same or more than isolated substances, thus suggesting a neuroprotective effect of the combination between *α*-LA and desvenlafaxine, given that AChE is a marker for degenerative diseases [105].

### 5.2. Human Studies

Regarding drugs for the treatment of AD, a clinical trial was performed in which a clinical and neuropsychological evaluation was conducted to assess the effects of *α*-LA combined conventional treatments. Participants were separated into two groups: the first group (A) were patients with AD and with type 2 diabetes mellitus (T2-DM2) and the second group (B) were patients with AD without T2-DM2. Patients received 600 mg/day of *α*-LA, and all maintained their treatments with acetylcholinesterase inhibitors, donepezil (5 to 10 mg/day), rivastigmine (6 to 12 mg/day), and galantamine (8 to 16 mg/day), as well as an NMDA receptor antagonist, memantine (10 to 20 mg/day). The results of the study showed that 44% of the first group and 41% of the second reported adverse reactions after the association of *α*-LA and the conventional treatment, including muscle cramps and gastrointestinal and sleep disorders. The findings of the neuropsychological evaluation showed that the general levels of dementia improved in the group of patients who had AD and T2-DM2 compared to the group with only AD at any moment of evaluation, which was performed three times: at the beginning of treatment, after 8 months, and after 16 months of follow-up [30].

## 6. Conclusions

Mitochondrial damage in the neurons of individuals with AD has been known for 25 years [39], and currently, more and more evidence points to mitochondria as a strong therapeutic target in several diseases associated with aging, including AD [43]. Oxidative damage caused by increased ROS in neurons is a consequence of a number of mechanisms associated with senescence, and the role of mitochondria seems to be crucial in the cascade of AD pathology events. Interventions for mitochondrial dysfunction have the potential to reduce cognitive decline in AD as a consequence of reestablishing glucose and lipid metabolism, calcium homeostasis, and the regulation of cell death signalling pathways [50]. In this sense, antioxidants, such as *α*-LA, are potential candidates in the strategy of restoring mitochondrial function, with evident benefits on mitobiogenesis, besides acting as a cofactor of mitochondrial enzymatic complexes—an essential pathway for energy production and metabolic regulation [87]—and directly scavenging ROS. In addition, *α*-LA shows effects on inflammasomes, on the one hand by reducing proinflammatory mediators such as IL-2, IFN-*γ*, and TNF-*α*, and on the other hand by increasing anti-inflammatory cytokines, such as IL-10. Additionally, the effects of *α*-LA show high coverage of sites of action, being considered a regulator of the expression of some genes, for example, the genes that code for nuclear factors Nrf2 and NF-*κ*B. Thus, *α*-LA is an agent with multiple actions on the cellular machinery, acting in several mechanisms that are involved in the pathology of AD. A large number of studies show the benefits of *α*-LA on symptoms of AD, but the amount of information about which pathways undergo direct or indirect interference of this antioxidant, now also considered neuroprotective, is still incipient. Clinical studies investigating *α*-LA in AD—limited to clinical parameters—focused primarily on brain areas associated with the symptoms of AD and did not investigate interactions, which occurred, at the molecular level. In contrast, studies that aimed to clarify the molecular mechanisms of interactions between *α*-LA and key molecules in the pathophysiology of AD have been conducted without having experimental groups treated with *α*-LA only, which is always associated with another antioxidant, to contrast with control groups. Another relevant aspect, but still considered very little in the current study of *α*-LA in AD, is the interaction between the drugs used in the treatment of AD and the supplementation of *α*-LA. Most studies that apply *α*-LA in parallel to treatment with standard drugs for AD do not present data on the possibility of pharmacokinetic or pharmacodynamic interactions, leaving a mechanism gap in the association response between these agents in the central nervous system. Even if the *α*-LA safety profile is well established, further studies should be conducted to precisely explain the effects, mainly in the brain. Additionally, isomers or a racemic mixture in AD models necessitate focus and investment in the development of improvements in molecular aspects of *α*-LA. These efforts could improve their pharmacokinetic profile once its low bioavailability and short half-life characteristics are widely known problems about *α*-LA. However, *α*-LA presents a neuroprotective and anti-inflammatory molecule profile with the ability to revert cellular damage in the central nervous system; thus, it is considered an epigenetic modulator in mechanisms associated with oxidative stress and inflammation. Therefore, *α*-LA protects against the progression or even the establishment of the toxic tissue environment resulting from the pathogenesis of AD. These characteristics make *α*-LA a nutraceutical with great potential for the treatment of this disease, as well as an agent with potential benefits for mitochondrial dysfunction. The ability to act on the reestablishment of mitochondrial function could block the progression or even reverse the damage to the brain tissue that occurs in AD.
